# A Case Report of Verheij Syndrome

**DOI:** 10.7759/cureus.66692

**Published:** 2024-08-12

**Authors:** Dhiran Sivasubramanian, Ahila Ayyavoo

**Affiliations:** 1 Critical Care Medicine, Christian Medical College, Vellore, IND; 2 Pediatric Endocrinology, G. Kuppuswamy Naidu Memorial Hospital, Coimbatore, IND

**Keywords:** turner's syndrome, whole exome sequencing (wes), noonan syndrome, 8q24 gene rearrangement, growth hormone therapy, genetic mutation, congenital short stature, puf60 gene, verheij syndrome

## Abstract

Verheij syndrome (VRJS) is a rare genetic disorder characterized by a range of developmental issues and physical abnormalities. This condition is caused by mutations or deletions in the PUF60 (poly-U-binding factor 60 kDa) gene, which is located on the long arm of chromosome 8, specifically in the q24.3 region. We present a unique case of an 11-year-old girl child with VRJS. The child presented with absence seizures. She was noted to have short stature, spina bifida of the lower cervical vertebrae, and a smaller right kidney on ultrasonography. This case expands the phenotypic spectrum of VRJS by demonstrating a milder presentation, highlighting the importance of a high index of suspicion for the diagnosis, even in atypical presentations. Whole exome sequencing identified the causative mutation, confirming the diagnosis. Growth hormone therapy was initiated for short stature but discontinued due to the subsequent development of idiopathic intracranial hypertension. Additionally, this report represents the first documented case of VRJS in India, emphasizing the importance of global data sharing and collaboration for improving the understanding and management of rare genetic disorders.

## Introduction

Verheij syndrome (VRJS) is a rare genetic disorder characterized by a spectrum of developmental and physical anomalies [1]. First described by Verheij et al. in 2009 [2], this condition results from deletion at the 8q24.3 chromosomal region. Recent studies identified pathogenic variants in the PUF60 (poly-U-binding factor 60 kDa) gene [1]. The pathophysiology of VRJS is the haploinsufficiency of PUF60 protein, a key player in RNA splicing and gene regulation [1]. In most cases, VRJS occurs sporadically during embryonic development, with no family history of the condition. However, in rare instances, it can be inherited in an autosomal dominant manner [1]. Patients commonly present with intellectual disability, distinctive facial features, spino-vertebral deformities, irido-retinal coloboma, cardiac defects, and renal abnormalities [1,3]. Differential diagnoses for VRJS include other genetic syndromes, particularly those involving congenital anomalies and developmental delays. Notably, Cornelia de Lange syndrome, Noonan syndrome, Coffin-Siris syndrome (CSS), and Rubinstein-Taybi syndrome (RSTS) are often considered due to similar physical characteristics [1,4,5]. Diagnosis of VRJS relies on a combination of clinical assessments and specialized genetic testing. A thorough physical examination and detailed family medical history form the initial evaluation. Genetic confirmation is done with array comparative genomic hybridization (array-CGH) and whole exome sequencing (WES) [1,6]. Management strategies for VRJS focus on mitigating symptoms and enhancing quality of life through a multidisciplinary approach. Although few patients have needed hormonal treatments, it's essential to evaluate the growth potential and hormonal status on an individual basis. Prompt recognition and treatment can enhance growth rates and improve overall health outcomes. We report an 11-year-old girl with a heterozygous mutation of PUF60, a pathogenic variant, highlighting the clinical features, the diagnostic process, and the management of a patient with VRJS. This is the first reported case of VRJS in India.

## Case presentation

An 11-year-old girl was born by cesarean section at term of an uneventful pregnancy with a birth weight of 2600 g, with a history of phototherapy for neonatal jaundice. She presented to the hospital with absence seizures, each lasting about one minute, followed by regaining consciousness. She experienced approximately four episodes in the last six months, with no history of bowel or bladder incontinence during the episodes. The patient was initially started on lamotrigine 25 mg BD for seizures but later switched to and currently on oxcarbazepine 300 mg BD. Her mother reported a history of a febrile seizure at two years of age.

She was referred to pediatric endocrinology for evaluation of short stature. Past medical records include a karyotyping done at one year of age showing 46 XX and an echocardiogram showing a patent ductus arteriosus with a left-to-right shunt and no other cardiac abnormalities. Her development was normal except for a mild delay in motor milestones and started to walk only by 18 months of age. Currently, she is in sixth grade, with an average school performance. There is no family history of seizures or genetic or neuromuscular developmental diseases, and the parents are unrelated.

**Figure 1:**
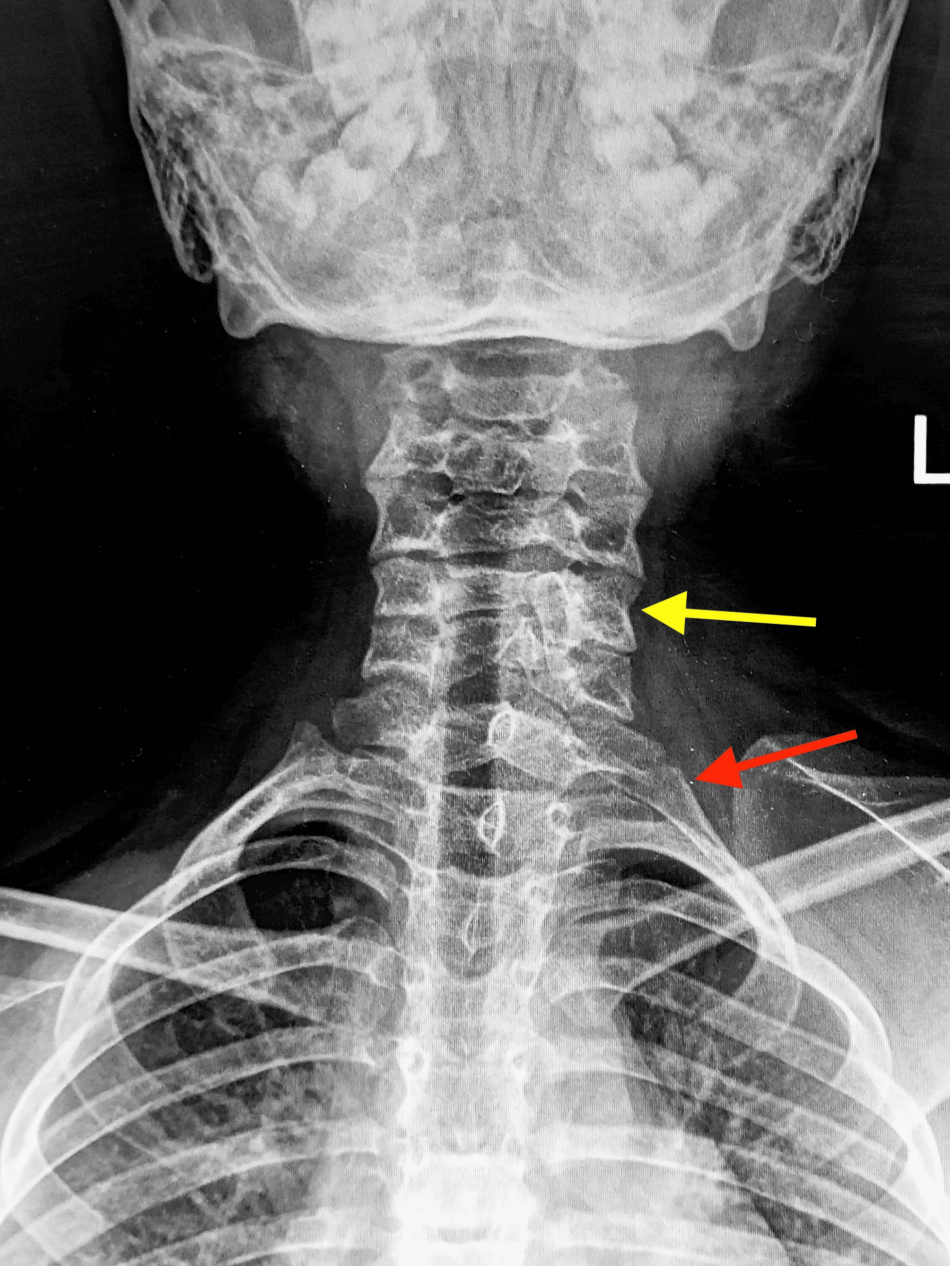
An anteroposterior radiograph of the cervical spine showing spina bifida of the C5 vertebrae (yellow arrow) and left cervical rib (red arrow)

Vitals on presentation included a heart rate of 100 beats/min, a blood pressure of 101/67 mmHg, a respiratory rate of 26/min, and an oxygen saturation of 96%. Her measured height was 129.5 cm with a Z score of -2.27 in height-for-age, and her weight was 36.7 kg with a Z score of -0.25 in weight-for-age according to WHO growth charts [7], with a bone age of nine years calculated using the Greulich and Pyle method [8]. The arm span was 126 cm, the upper/lower segment ratio was 0.9, and the total body surface area was 1.14 m^2^. Physical examination revealed a short neck, a skin tag on the anterior chest wall, and a café-au-lait spot on the right upper abdomen. Clinical examination of cardiovascular, respiratory, abdominal, and nervous systems were normal. Routine blood investigations were within the reference range (Table 1). Thyroid profile, liver and renal function tests, serum electrolytes, and serum calcium were normal. An ultrasonography of the abdomen showed a smaller right kidney compared to the left and the uterus deviated more towards the right side. The echocardiogram at presentation showed good biventricular function and spontaneously closed patent ductus arteriosus. A cervical spine radiograph revealed spina bifida of the fifth cervical vertebrae and a cervical rib (Figure 1). A video electroencephalogram recorded was within normal limits. Magnetic resonance imaging (MRI) of the brain showed no abnormalities. Early differentials for the short stature with short neck were Turner syndrome and Noonan syndrome; the possibility of Turner was ruled out as the karyotype was 46 XX. Based on clinical suspicion of Noonan syndrome [9], WES was performed, revealing a mutation in the PUF60 gene at exon 10, a heterozygous likely pathogenic variant that results in VRJS.

**Table 1:** Hematological investigations ALP: alkaline phosphatase; ALT: alanine aminotransferase; T3: triiodothyronine; T4: thyroxine; TSH: thyroid-stimulating hormone; IGF-1: insulin-like growth factor; PTH: parathyroid hormone

Type	Patient value	Reference value	Units
ALP	193	Less than 350	IU/L
ALT	26	10-40	IU/L
Bicarbonate	29	22-29	mEq/L
Calcium	9.8	8.8-10.8	mg/dL
Chloride	102	90-110	mEq/L
Phosphorus	4.2	4.5-6.5	mg/dL
Potassium	3.5	3.4-4.7	mEq/L
Sodium	140	135-145	mEq/L
Hemoglobin	12	10-15.5	g/dL
Platelet	308,000	150,000-400,000	/mm^3^
White blood cell total count	7200	4,800-10,800	/mm^3^
T3, total	188	68-186	ng/dL
T4, total	10.9	5-12	ng/dL
TSH	2	0.55-5.31	µU/mL
Anti-Müllerian hormone	1.06	1.0-4.0	ng/mL
IGF-1	129	58-465	ng/mL
PTH	53.3	14-65	pg/mL

As the patient's height was less than two standard deviations below the mean for her age, a sex steroid-primed growth hormone (GH) stimulation test was conducted, showing subnormal peak values, warranting a need for GH therapy [10]. After a detailed explanation of the treatment, its effects, and its potential side effects, the child was initiated on daily subcutaneous injections of human GH 3.3 units equivalent to 28 mcg/kg/day at bedtime. Routine monitoring of thyroid profile and insulin-like growth factor (IGF-1) levels was conducted. The patient gained 1.8 cm after three months of therapy, with an IGF-1 level of 623 ng/ml. The patient had complaints of a new-onset headache, which progressively worsened over a week. No focal neurological deficits were observed on examination. An MRI of the brain showed features of idiopathic intracranial hypertension. Due to raised intracranial pressure, GH therapy was discontinued [11], and her symptoms resolved. The patient was reviewed six months later and had gained 3.2 cm in height after stopping GH therapy. The patient continues to be monitored with regular follow-ups.

## Discussion

Common anomalies seen in VRJS include skeletal abnormalities in 80% of cases, ocular malformations in 70%, intellectual disabilities in 60%, and cardiac problems in 40% [12,13]. Less common manifestations include renal and audiologic defects, cervical spine segmentation defects, and growth impairment resulting in short stature. Ocular manifestations, such as irido-retinal or chorio-retinal coloboma, are important diagnostic clues [13].

Our patient presented with several clinical features suggestive of VRJS, including short stature, with her height below two standard deviations of the mean for her age, developmental delay in motor milestones, a spine radiograph showing spina bifida of the lower cervical vertebrae, an early echocardiogram showing a patent ductus arteriosus, an abdominal ultrasound showing a smaller right kidney, and frequent episodes of absence seizures. Seizures were one of the major presentations of VRJS in the literature [2,3,12].

This highlights the importance of a wide phenotypic heterogeneity of this syndrome, and the need to maintain a low threshold of doubt to start a diagnostic workup, even if not all the features are seen in a patient. The reason behind the vast presentation spectrum of VRJS is the pleiotropy of the gene [12,13] and also that the amount of genetic material lost in every individual mutation is unique, resulting in a different presentation in every case.

A study published in February 2024 reports only 73 cases of VRJS in literature to date [14]. This rare craniofacial spliceosomopathy arises due to a microdeletion of the 8q24.3 chromosomal region [13]. Further research pointed out that a mutation specifically in PUF60 on chromosome 8q24.3 is the causative factor of VRJS [10]. PUF60 encodes a protein (spliceosome) involved in the recognition of 3′ splice-site and the regulation of transcription. Deletion of PUF60 results in frameshift mutations, resulting in premature stop codons [1]. Milder forms can be caused by the presence of pathogenic variants of the PUF60 gene, as seen in our case. This mutation can be caused sporadically or can be inherited from parents. To date, there is only one reported case of inherited VRJS in literature [12]. Different variants of mutations have been identified including splice-site, missense, and non-sense mutations [3,15].

Treatment depends on the presentation. The best approach is multidisciplinary management, involving experts from different specialties along with speech, language, physical, and occupational therapy. Early surveillance for growth retardation is warranted, as hormone therapy is indicated to reach normal adult growth and maintain bone health. In our case, growth hormone therapy could not be continued due to adverse effects. Symptomatic management and a multimodal approach can sometimes lead to polypharmacy and undesirable side effects, leading to non-compliance and poor response to treatment. Hence, further research into alternative treatment modalities that target the root cause of the syndrome needs to be identified.

## Conclusions

This case report highlights a new pathogenic variant in the PUF60 gene associated with VRJS, marking the first documented instance of this syndrome in India. The patient's presentation was notably milder compared to the more severe manifestations often reported in the literature, which underscores the phenotypic variability of this rare genetic disorder. Reporting this case from India highlights the importance of global data sharing and collaboration to improve outcomes for patients with rare genetic disorders.
